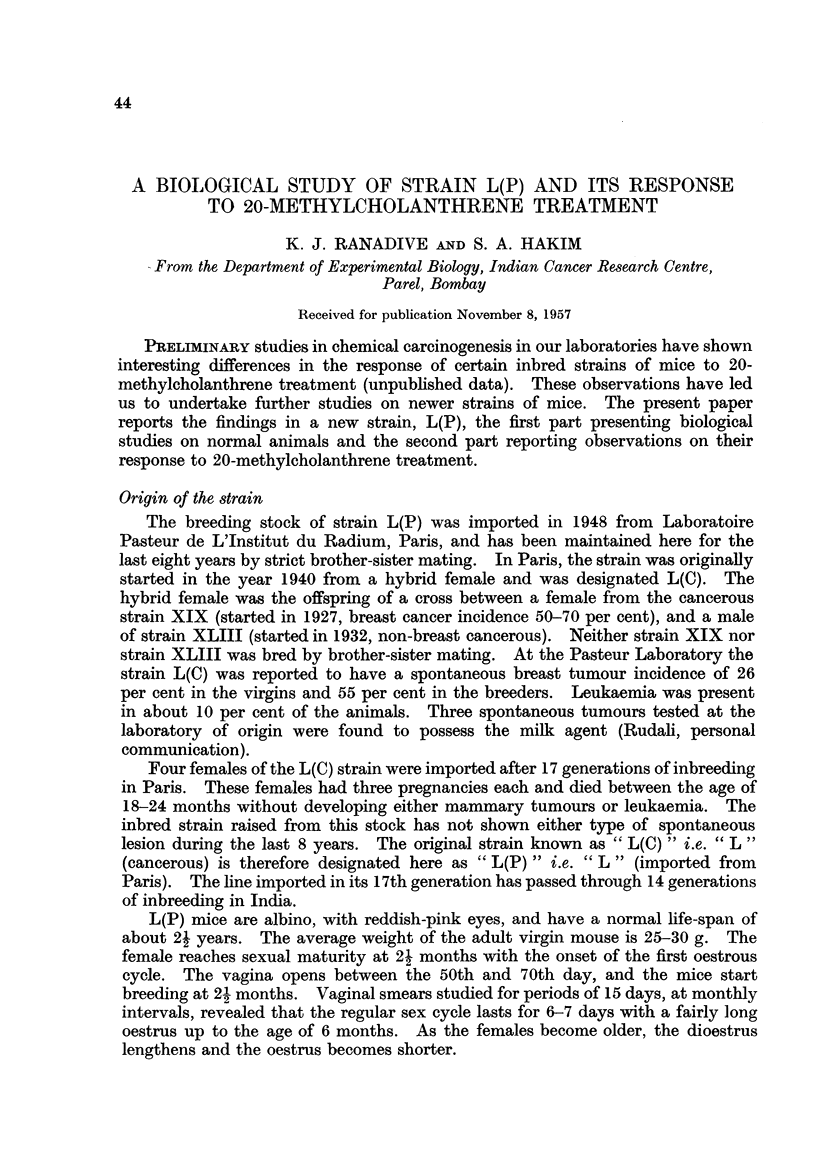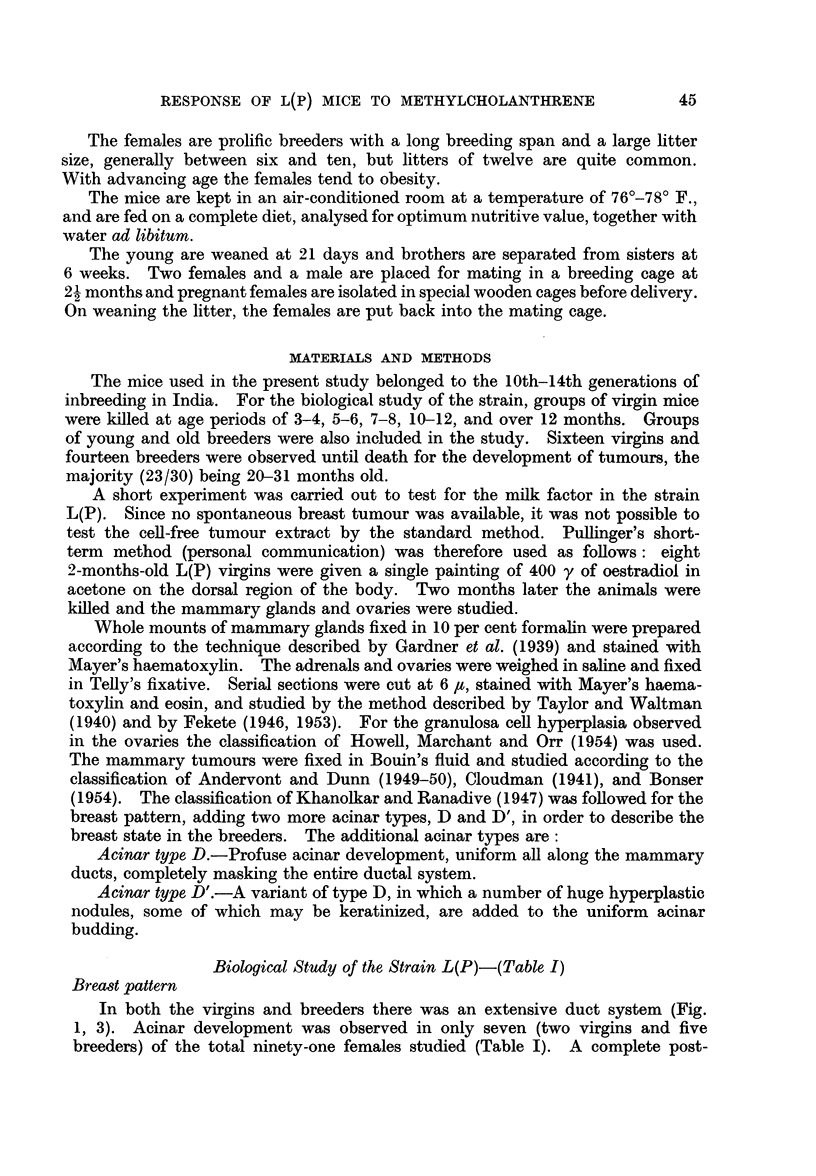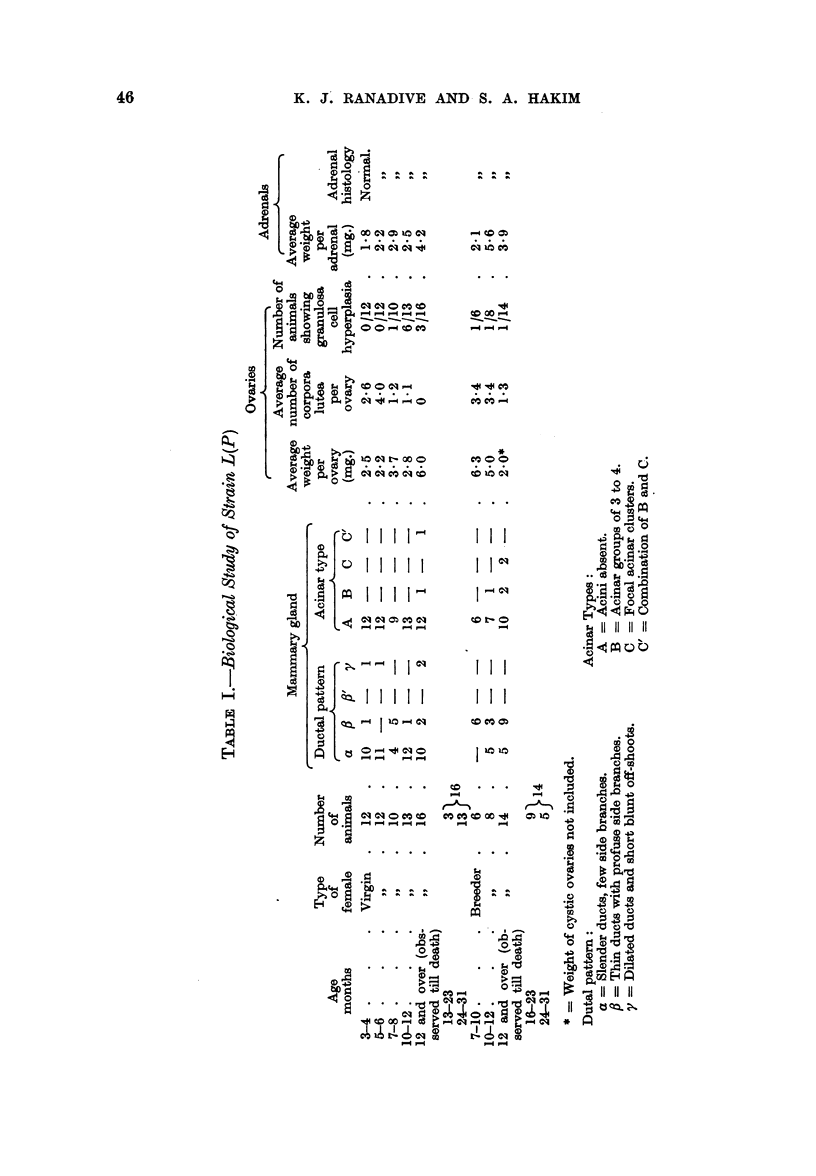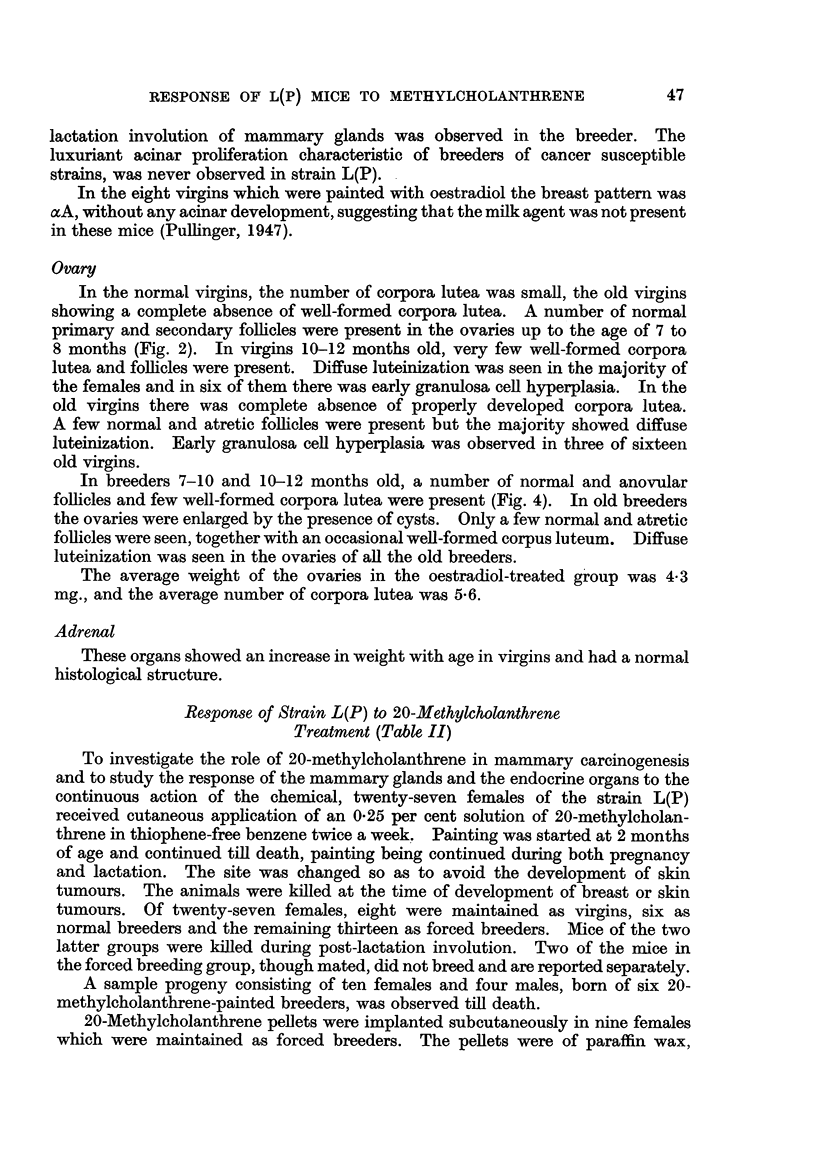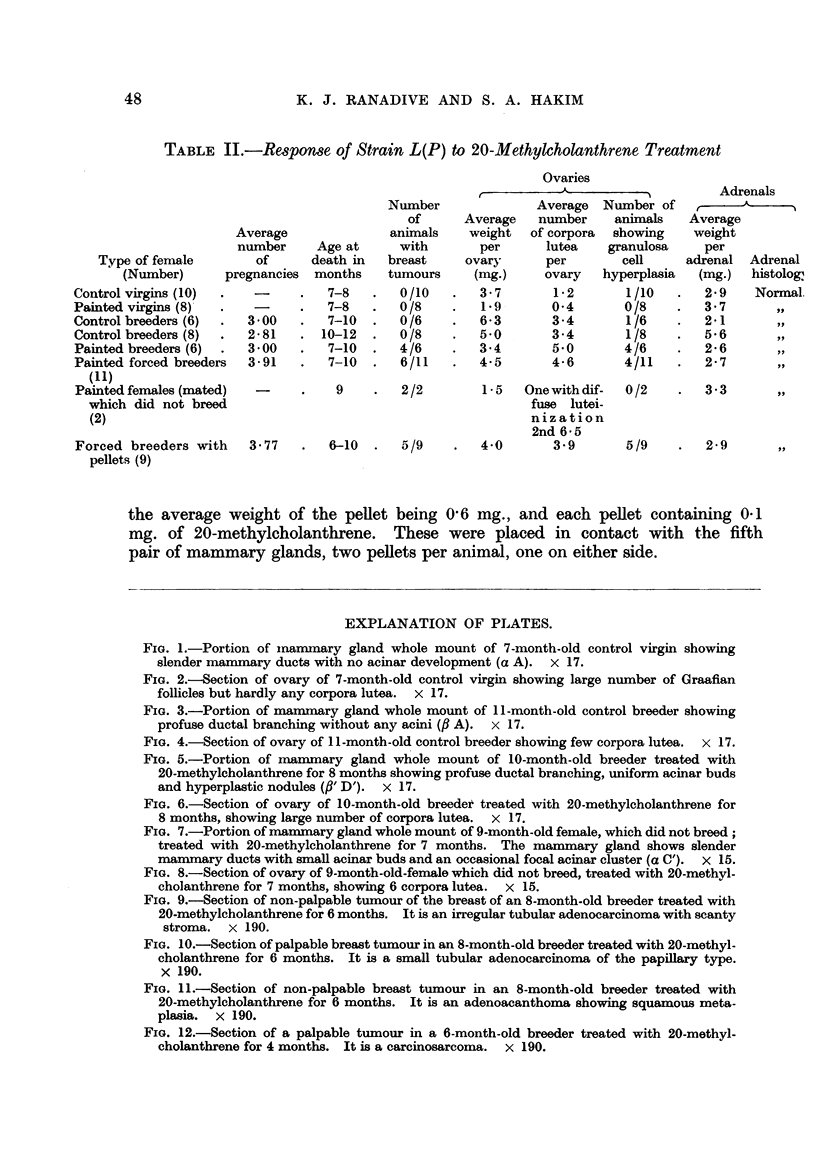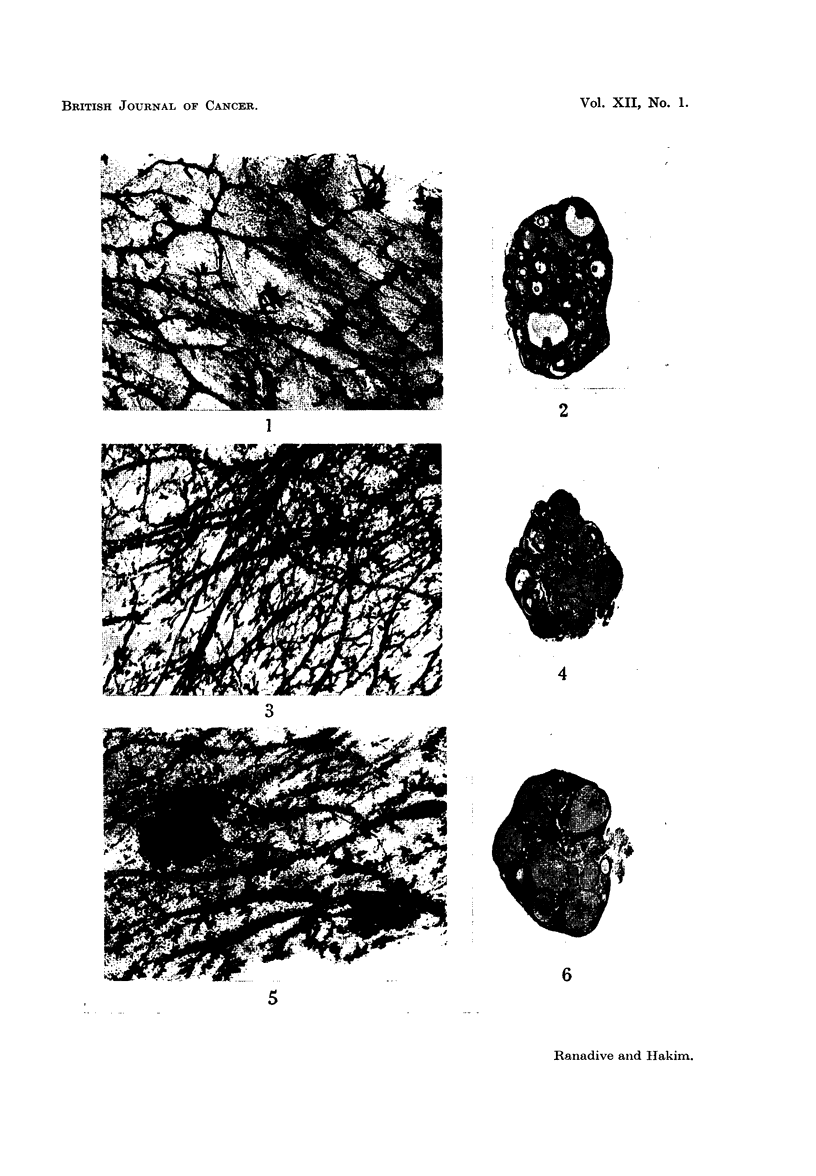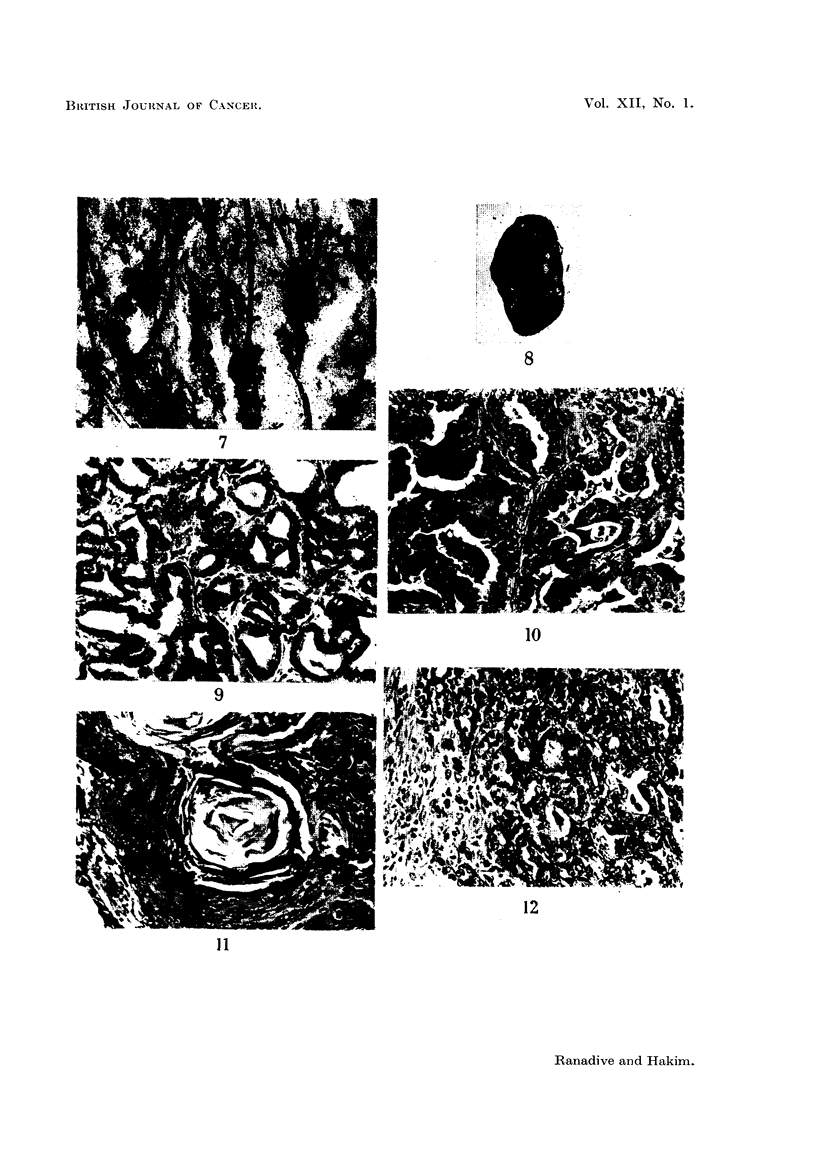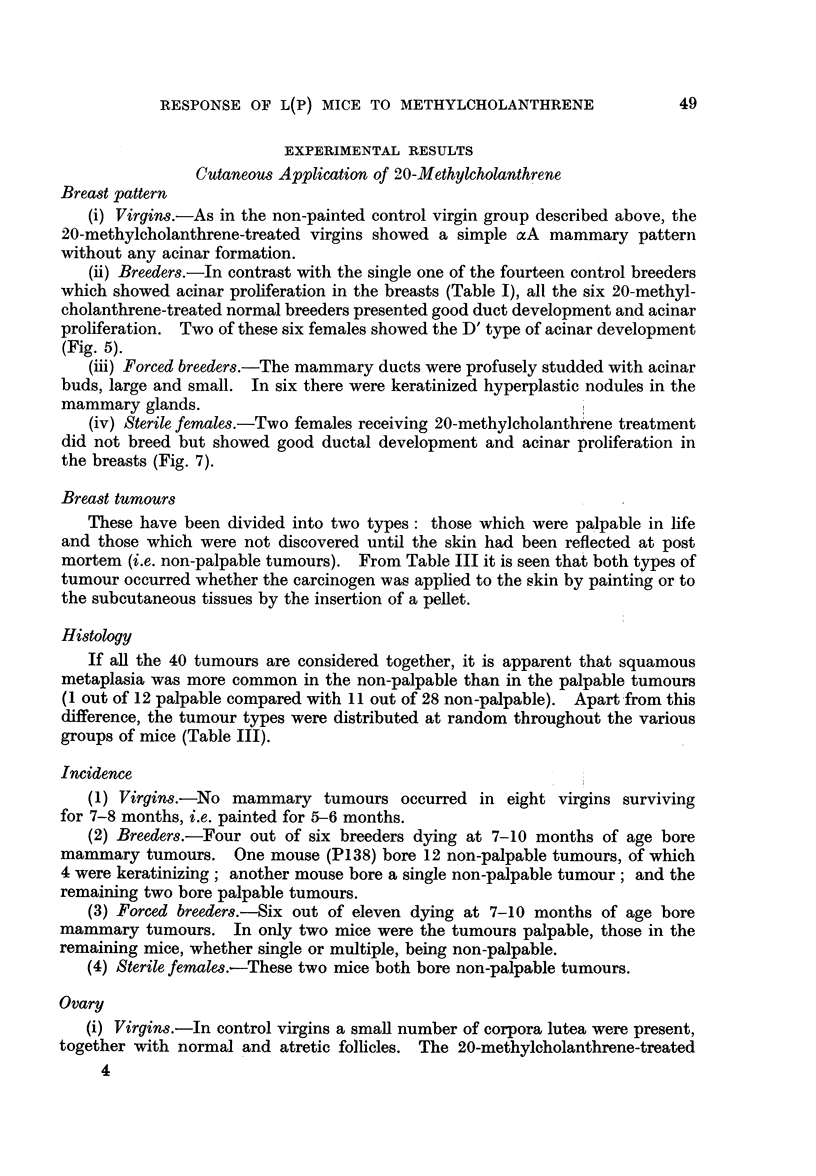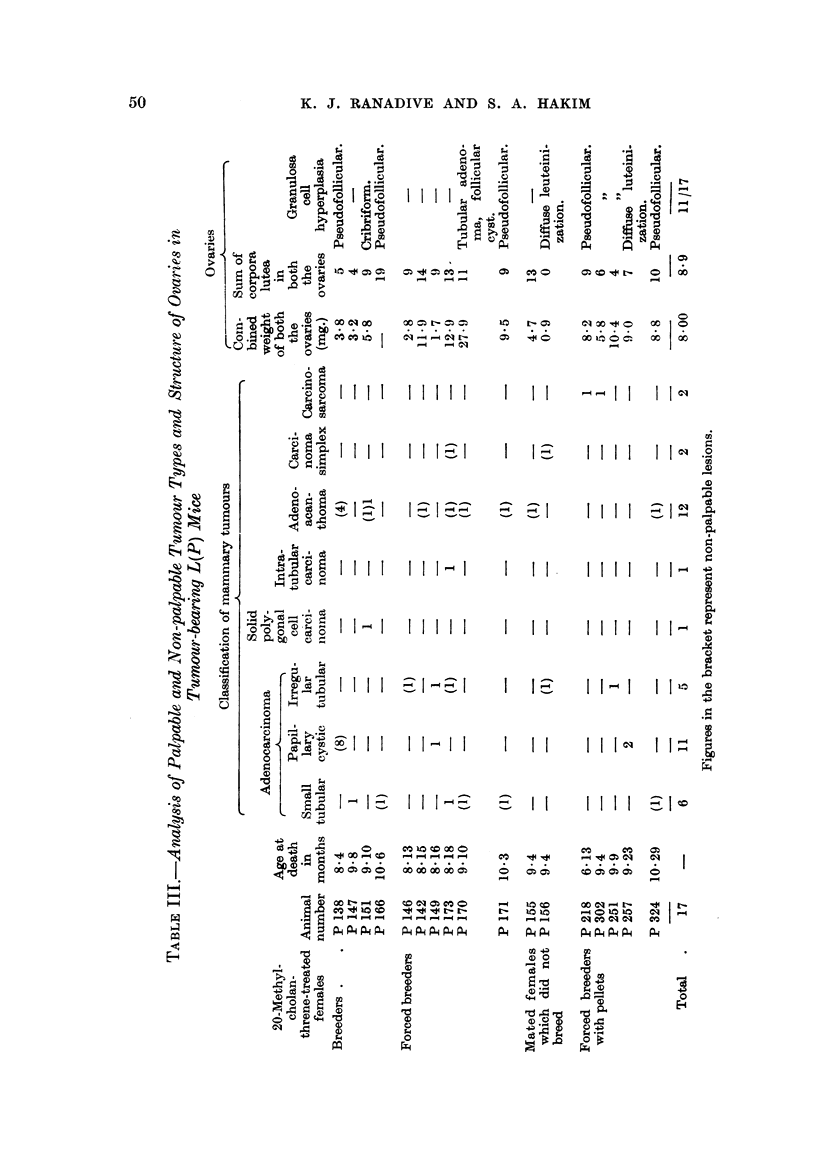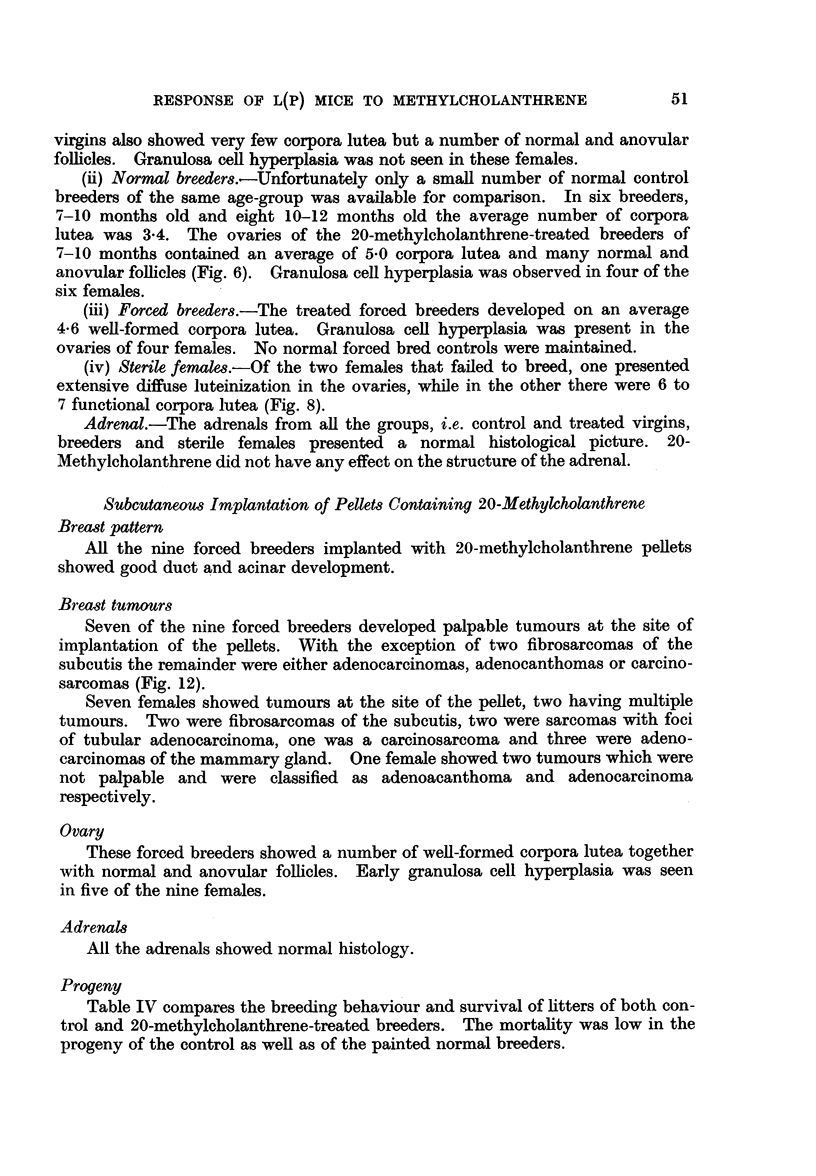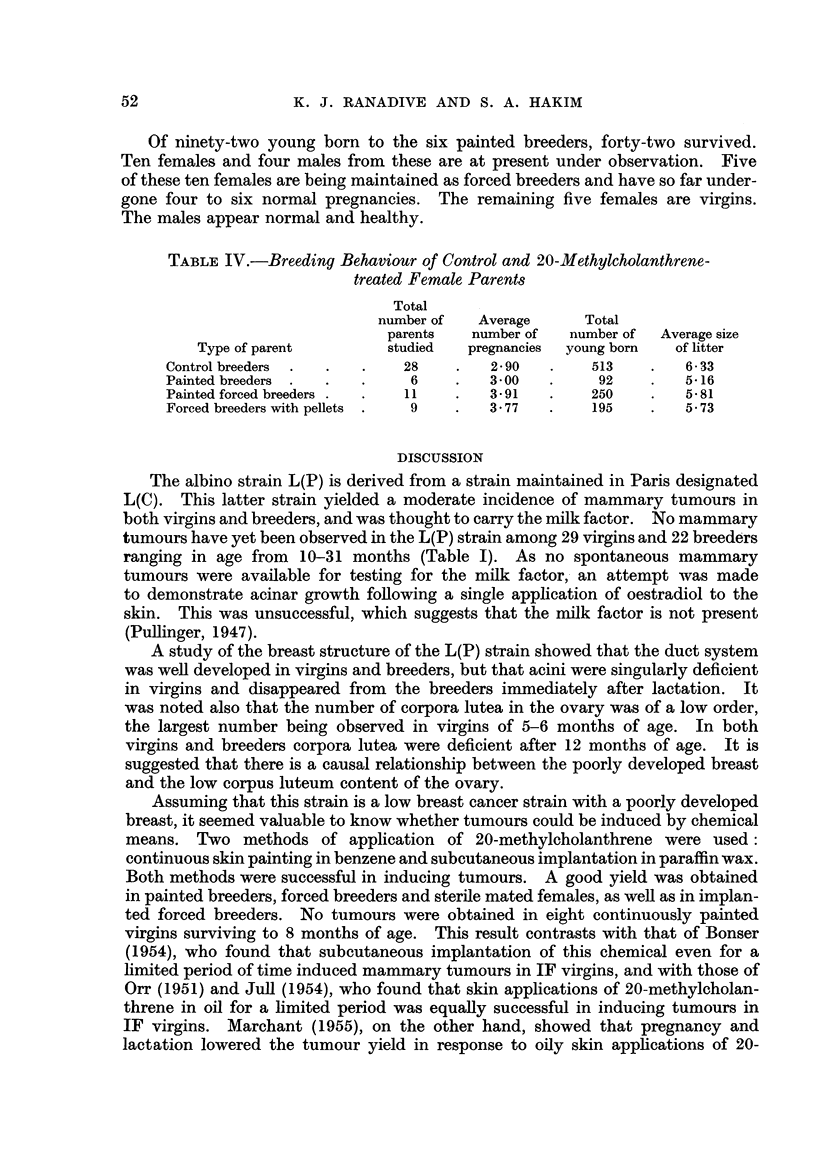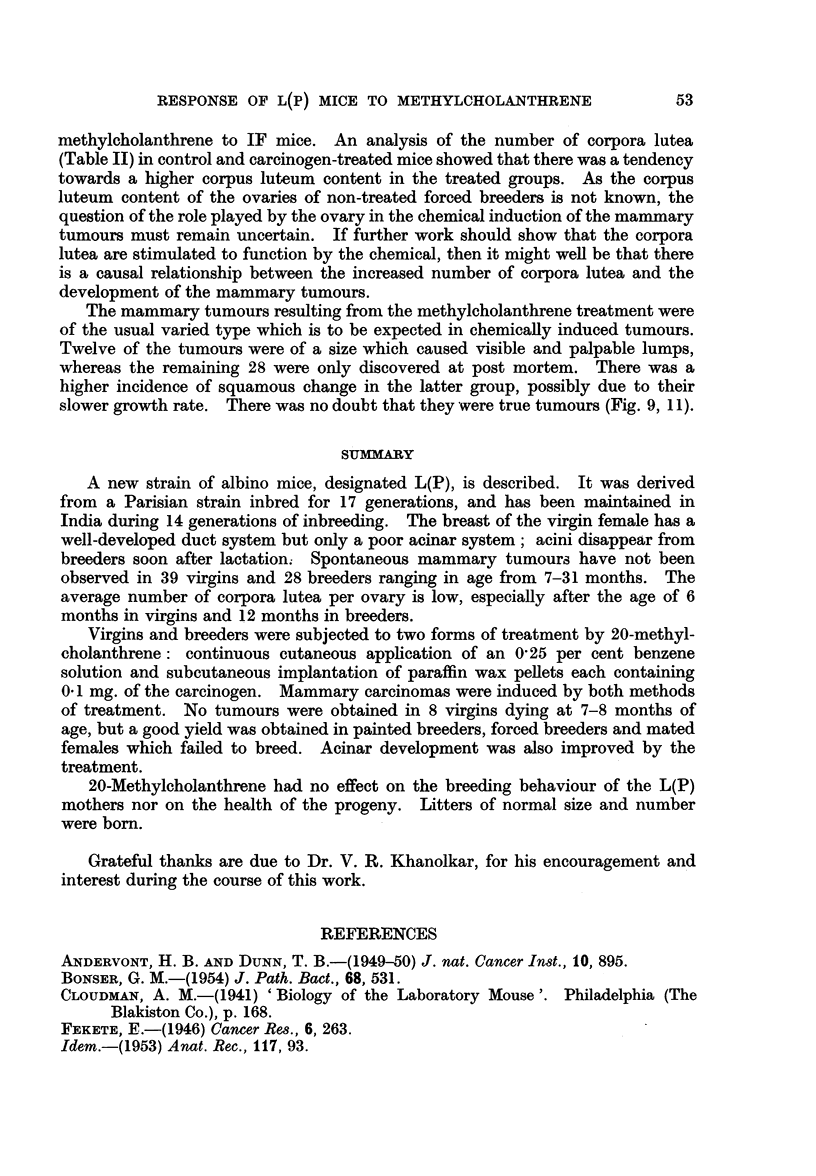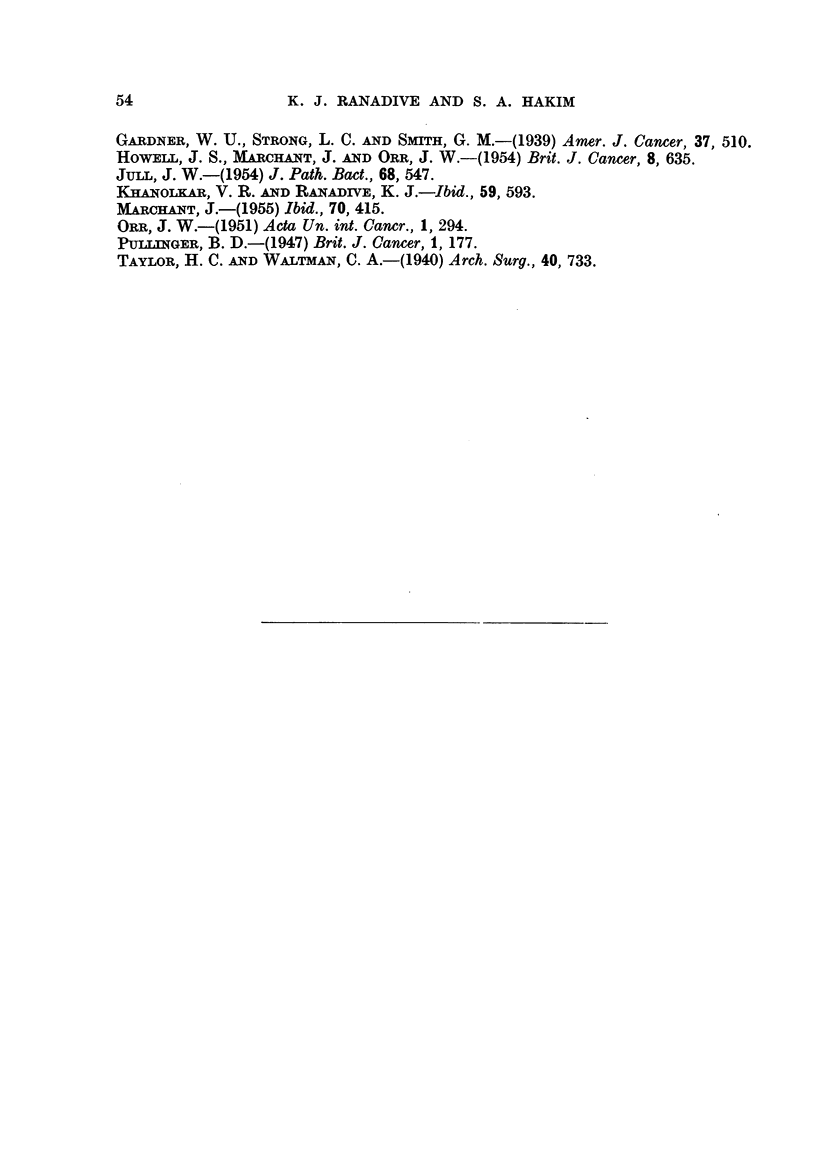# A Biological Study of Strain L(P) and its Response to 20-Methylcholanthrene Treatment

**DOI:** 10.1038/bjc.1958.7

**Published:** 1958-03

**Authors:** K. J. Ranadive, S. A. Hakim

## Abstract

**Images:**


### A BIOLOGICAL STUDY OF STRAIN L(P) AND ITS RESPONSE TO 20-METHYLCHOLANTHRENE TREATMENT

K. J. RANADIVE AND S. A. HAKIM

*From the Department of Experimental Biology, Indian Cancer Research Centre, Parel, Bombay*

Received for publication November 8, 1957

PRELIMINARY studies in chemical carcinogenesis in our laboratories have shown interesting differences in the response of certain inbred strains of mice to 20-methylcholanthrene treatment (unpublished data). These observations have led us to undertake further studies on newer strains of mice. The present paper reports the findings in a new strain, L(P), the first part presenting biological studies on normal animals and the second part reporting observations on their response to 20-methylcholanthrene treatment.

*Origin of the strain*

The breeding stock of strain L(P) was imported in 1948 from Laboratoire Pasteur de L'Institut du Radium, Paris, and has been maintained here for the last eight years by strict brother-sister mating. In Paris, the strain was originally started in the year 1940 from a hybrid female and was designated L(C). The hybrid female was the offspring of a cross between a female from the cancerous strain XIX (started in 1927, breast cancer incidence 50–70 per cent), and a male of strain XLIII (started in 1932, non-breast cancerous). Neither strain XIX nor strain XLIII was bred by brother-sister mating. At the Pasteur Laboratory the strain L(C) was reported to have a spontaneous breast tumour incidence of 26 per cent in the virgins and 55 per cent in the breeders. Leukaemia was present in about 10 per cent of the animals. Three spontaneous tumours tested at the laboratory of origin were found to possess the milk agent (Rudali, personal communication).

Four females of the L(C) strain were imported after 17 generations of inbreeding in Paris. These females had three pregnancies each and died between the age of 18–24 months without developing either mammary tumours or leukaemia. The inbred strain raised from this stock has not shown either type of spontaneous lesion during the last 8 years. The original strain known as "L(C)" *i.e.* "L" (cancerous) is therefore designated here as "L(P)" *i.e.* "L" (imported from Paris). The line imported in its 17th generation has passed through 14 generations of inbreeding in India.

L(P) mice are albino, with reddish-pink eyes, and have a normal life-span of about 2½ years. The average weight of the adult virgin mouse is 25–30 g. The female reaches sexual maturity at 2½ months with the onset of the first oestrous cycle. The vagina opens between the 50th and 70th day, and the mice start breeding at 2½ months. Vaginal smears studied for periods of 15 days, at monthly intervals, revealed that the regular sex cycle lasts for 6–7 days with a fairly long oestrus up to the age of 6 months. As the females become older, the dioestrus lengthens and the oestrus becomes shorter.

The females are prolific breeders with a long breeding span and a large litter size, generally between six and ten, but litters of twelve are quite common. With advancing age the females tend to obesity.

The mice are kept in an air-conditioned room at a temperature of 76°–78° F., and are fed on a complete diet, analysed for optimum nutritive value, together with water *ad libitum*.

The young are weaned at 21 days and brothers are separated from sisters at 6 weeks. Two females and a male are placed for mating in a breeding cage at 2½ months and pregnant females are isolated in special wooden cages before delivery. On weaning the litter, the females are put back into the mating cage.

### MATERIALS AND METHODS

The mice used in the present study belonged to the 10th–14th generations of inbreeding in India. For the biological study of the strain, groups of virgin mice were killed at age periods of 3–4, 5–6, 7–8, 10–12, and over 12 months. Groups of young and old breeders were also included in the study. Sixteen virgins and fourteen breeders were observed until death for the development of tumours, the majority (23/30) being 20–31 months old.

A short experiment was carried out to test for the milk factor in the strain L(P). Since no spontaneous breast tumour was available, it was not possible to test the cell-free tumour extract by the standard method. Pullinger's short-term method (personal communication) was therefore used as follows: eight 2-months-old L(P) virgins were given a single painting of 400 $\gamma$ of oestradiol in acetone on the dorsal region of the body. Two months later the animals were killed and the mammary glands and ovaries were studied.

Whole mounts of mammary glands fixed in 10 per cent formalin were prepared according to the technique described by Gardner *et al.* (1939) and stained with Mayer's haematoxylin. The adrenals and ovaries were weighed in saline and fixed in Telly's fixative. Serial sections were cut at 6 $\mu$, stained with Mayer's haematoxylin and eosin, and studied by the method described by Taylor and Waltman (1940) and by Fekete (1946, 1953). For the granulosa cell hyperplasia observed in the ovaries the classification of Howell, Marchant and Orr (1954) was used. The mammary tumours were fixed in Bouin's fluid and studied according to the classification of Andervont and Dunn (1949–50), Cloudman (1941), and Bonser (1954). The classification of Khanolkar and Ranadive (1947) was followed for the breast pattern, adding two more acinar types, D and D′, in order to describe the breast state in the breeders. The additional acinar types are:

*Acinar type D.*—Profuse acinar development, uniform all along the mammary ducts, completely masking the entire ductal system.

*Acinar type D′.*—A variant of type D, in which a number of huge hyperplastic nodules, some of which may be keratinized, are added to the uniform acinar budding.

### *Biological Study of the Strain L(P)—(Table I)*

#### *Breast pattern*

In both the virgins and breeders there was an extensive duct system (Fig. 1, 3). Acinar development was observed in only seven (two virgins and five breeders) of the total ninety-one females studied (Table I). A complete post-

TABLE I.—*Biological Study of Strain L(P)*

| Age months | Type of female | Number of animals | Mammary gland: Ductal pattern α | β | β′ | γ | Mammary gland: Acinar type A | B | C | C′ | Ovaries: Average weight per ovary (mg.) | Ovaries: Average number of corpora lutea per ovary | Ovaries: Number of animals showing granulosa cell hyperplasia | Adrenals: Average weight per adrenal (mg.) | Adrenals: Adrenal histology |
|---|---|---|---|---|---|---|---|---|---|---|---|---|---|---|---|
| 3–4 | Virgin | 12 | 10 | 1 | — | 1 | 12 | — | — | — | 2·5 | 2·6 | 0/12 | 1·8 | Normal. |
| 5–6 | ,, | 12 | 11 | — | — | 1 | 12 | — | — | — | 2·2 | 4·0 | 0/12 | 2·2 | ,, |
| 7–8 | ,, | 10 | 4 | 5 | — | — | 9 | — | — | — | 3·7 | 1·2 | 1/10 | 2·9 | ,, |
| 10–12 | ,, | 13 | 12 | 1 | — | — | 13 | — | — | — | 2·8 | 1·1 | 6/13 | 2·5 | ,, |
| 12 and over (observed till death) | ,, | 16 | 10 | 2 | — | 2 | 12 | 1 | — | 1 | 6·0 | 0 | 3/16 | 4·2 | ,, |
| 13–23 | | 3 } 16 | | | | | | | | | | | | | |
| 24–31 | | 13 } | | | | | | | | | | | | | |
| 7–10 | Breeder | 6 | — | 6 | — | — | 6 | — | — | — | 6·3 | 3·4 | 1/6 | 2·1 | ,, |
| 10–12 | ,, | 8 | 5 | 3 | — | — | 7 | 1 | — | — | 5·0 | 3·4 | 1/8 | 5·6 | ,, |
| 12 and over (observed till death) | ,, | 14 | 5 | 9 | — | — | 10 | 2 | 2 | — | 2·0* | 1·3 | 1/14 | 3·9 | ,, |
| 16–23 | | 9 } 14 | | | | | | | | | | | | | |
| 24–31 | | 5 } | | | | | | | | | | | | | |

* = Weight of cystic ovaries not included.

Dutal pattern :
α = Slender ducts, few side branches.
β = Thin ducts with profuse side branches.
γ = Dilated ducts and short blunt off-shoots.

Acinar Types :

A = Acini absent.

B = Acinar groups of 3 to 4.

C = Focal acinar clusters.

C′ = Combination of B and C.

lactation involution of mammary glands was observed in the breeder. The luxuriant acinar proliferation characteristic of breeders of cancer susceptible strains, was never observed in strain L(P).

In the eight virgins which were painted with oestradiol the breast pattern was $\alpha$A, without any acinar development, suggesting that the milk agent was not present in these mice (Pullinger, 1947).

*Ovary*

In the normal virgins, the number of corpora lutea was small, the old virgins showing a complete absence of well-formed corpora lutea. A number of normal primary and secondary follicles were present in the ovaries up to the age of 7 to 8 months (Fig. 2). In virgins 10–12 months old, very few well-formed corpora lutea and follicles were present. Diffuse luteinization was seen in the majority of the females and in six of them there was early granulosa cell hyperplasia. In the old virgins there was complete absence of properly developed corpora lutea. A few normal and atretic follicles were present but the majority showed diffuse luteinization. Early granulosa cell hyperplasia was observed in three of sixteen old virgins.

In breeders 7–10 and 10–12 months old, a number of normal and anovular follicles and few well-formed corpora lutea were present (Fig. 4). In old breeders the ovaries were enlarged by the presence of cysts. Only a few normal and atretic follicles were seen, together with an occasional well-formed corpus luteum. Diffuse luteinization was seen in the ovaries of all the old breeders.

The average weight of the ovaries in the oestradiol-treated group was 4·3 mg., and the average number of corpora lutea was 5·6.

*Adrenal*

These organs showed an increase in weight with age in virgins and had a normal histological structure.

*Response of Strain L(P) to 20-Methylcholanthrene Treatment (Table II)*

To investigate the role of 20-methylcholanthrene in mammary carcinogenesis and to study the response of the mammary glands and the endocrine organs to the continuous action of the chemical, twenty-seven females of the strain L(P) received cutaneous application of an 0·25 per cent solution of 20-methylcholanthrene in thiophene-free benzene twice a week. Painting was started at 2 months of age and continued till death, painting being continued during both pregnancy and lactation. The site was changed so as to avoid the development of skin tumours. The animals were killed at the time of development of breast or skin tumours. Of twenty-seven females, eight were maintained as virgins, six as normal breeders and the remaining thirteen as forced breeders. Mice of the two latter groups were killed during post-lactation involution. Two of the mice in the forced breeding group, though mated, did not breed and are reported separately.

A sample progeny consisting of ten females and four males, born of six 20-methylcholanthrene-painted breeders, was observed till death.

20-Methylcholanthrene pellets were implanted subcutaneously in nine females which were maintained as forced breeders. The pellets were of paraffin wax,

TABLE II.—*Response of Strain L(P) to 20-Methylcholanthrene Treatment*

| Type of female (Number) | Average number of pregnancies | Age at death in months | Number of animals with breast tumours | Ovaries: Average weight per ovary (mg.) | Ovaries: Average number of corpora lutea per ovary | Ovaries: Number of animals showing granulosa cell hyperplasia | Adrenals: Average weight per adrenal (mg.) | Adrenals: Adrenal histology |
|---|---|---|---|---|---|---|---|---|
| Control virgins (10) | — | 7–8 | 0/10 | 3·7 | 1·2 | 1/10 | 2·9 | Normal. |
| Painted virgins (8) | — | 7–8 | 0/8 | 1·9 | 0·4 | 0/8 | 3·7 | „ |
| Control breeders (6) | 3·00 | 7–10 | 0/6 | 6·3 | 3·4 | 1/6 | 2·1 | „ |
| Control breeders (8) | 2·81 | 10–12 | 0/8 | 5·0 | 3·4 | 1/8 | 5·6 | „ |
| Painted breeders (6) | 3·00 | 7–10 | 4/6 | 3·4 | 5·0 | 4/6 | 2·6 | „ |
| Painted forced breeders (11) | 3·91 | 7–10 | 6/11 | 4·5 | 4·6 | 4/11 | 2·7 | „ |
| Painted females (mated) which did not breed (2) | — | 9 | 2/2 | 1·5 | One with diffuse luteinization 2nd 6·5 | 0/2 | 3·3 | „ |
| Forced breeders with pellets (9) | 3·77 | 6–10 | 5/9 | 4·0 | 3·9 | 5/9 | 2·9 | „ |

the average weight of the pellet being 0·6 mg., and each pellet containing 0·1 mg. of 20-methylcholanthrene. These were placed in contact with the fifth pair of mammary glands, two pellets per animal, one on either side.

---

### EXPLANATION OF PLATES.

FIG. 1.—Portion of mammary gland whole mount of 7-month-old control virgin showing slender mammary ducts with no acinar development (α A). × 17.

FIG. 2.—Section of ovary of 7-month-old control virgin showing large number of Graafian follicles but hardly any corpora lutea. × 17.

FIG. 3.—Portion of mammary gland whole mount of 11-month-old control breeder showing profuse ductal branching without any acini (β A). × 17.

FIG. 4.—Section of ovary of 11-month-old control breeder showing few corpora lutea. × 17.

FIG. 5.—Portion of mammary gland whole mount of 10-month-old breeder treated with 20-methylcholanthrene for 8 months showing profuse ductal branching, uniform acinar buds and hyperplastic nodules (β′ D′). × 17.

FIG. 6.—Section of ovary of 10-month-old breeder treated with 20-methylcholanthrene for 8 months, showing large number of corpora lutea. × 17.

FIG. 7.—Portion of mammary gland whole mount of 9-month-old female, which did not breed; treated with 20-methylcholanthrene for 7 months. The mammary gland shows slender mammary ducts with small acinar buds and an occasional focal acinar cluster (α C′). × 15.

FIG. 8.—Section of ovary of 9-month-old-female which did not breed, treated with 20-methylcholanthrene for 7 months, showing 6 corpora lutea. × 15.

FIG. 9.—Section of non-palpable tumour of the breast of an 8-month-old breeder treated with 20-methylcholanthrene for 6 months. It is an irregular tubular adenocarcinoma with scanty stroma. × 190.

FIG. 10.—Section of palpable breast tumour in an 8-month-old breeder treated with 20-methylcholanthrene for 6 months. It is a small tubular adenocarcinoma of the papillary type. × 190.

FIG. 11.—Section of non-palpable breast tumour in an 8-month-old breeder treated with 20-methylcholanthrene for 6 months. It is an adenoacanthoma showing squamous metaplasia. × 190.

FIG. 12.—Section of a palpable tumour in a 6-month-old breeder treated with 20-methylcholanthrene for 4 months. It is a carcinosarcoma. × 190.

1

2

3

4

5

6

7

8

9

10

11

12

### EXPERIMENTAL RESULTS

### *Cutaneous Application of* 20-*Methylcholanthrene*

*Breast pattern*

(i) *Virgins.*—As in the non-painted control virgin group described above, the 20-methylcholanthrene-treated virgins showed a simple $\alpha$A mammary pattern without any acinar formation.

(ii) *Breeders.*—In contrast with the single one of the fourteen control breeders which showed acinar proliferation in the breasts (Table I), all the six 20-methylcholanthrene-treated normal breeders presented good duct development and acinar proliferation. Two of these six females showed the D′ type of acinar development (Fig. 5).

(iii) *Forced breeders.*—The mammary ducts were profusely studded with acinar buds, large and small. In six there were keratinized hyperplastic nodules in the mammary glands.

(iv) *Sterile females.*—Two females receiving 20-methylcholanthrene treatment did not breed but showed good ductal development and acinar proliferation in the breasts (Fig. 7).

*Breast tumours*

These have been divided into two types : those which were palpable in life and those which were not discovered until the skin had been reflected at post mortem (*i.e.* non-palpable tumours). From Table III it is seen that both types of tumour occurred whether the carcinogen was applied to the skin by painting or to the subcutaneous tissues by the insertion of a pellet.

*Histology*

If all the 40 tumours are considered together, it is apparent that squamous metaplasia was more common in the non-palpable than in the palpable tumours (1 out of 12 palpable compared with 11 out of 28 non-palpable). Apart from this difference, the tumour types were distributed at random throughout the various groups of mice (Table III).

*Incidence*

(1) *Virgins.*—No mammary tumours occurred in eight virgins surviving for 7–8 months, *i.e.* painted for 5–6 months.

(2) *Breeders.*—Four out of six breeders dying at 7–10 months of age bore mammary tumours. One mouse (P138) bore 12 non-palpable tumours, of which 4 were keratinizing ; another mouse bore a single non-palpable tumour ; and the remaining two bore palpable tumours.

(3) *Forced breeders.*—Six out of eleven dying at 7–10 months of age bore mammary tumours. In only two mice were the tumours palpable, those in the remaining mice, whether single or multiple, being non-palpable.

(4) *Sterile females.*—These two mice both bore non-palpable tumours.

*Ovary*

(i) *Virgins.*—In control virgins a small number of corpora lutea were present, together with normal and atretic follicles. The 20-methylcholanthrene-treated

TABLE III.—*Analysis of Palpable and Non-palpable Tumour Types and Structure of Ovaries in Tumour-bearing L(P) Mice*

| | | | Classification of mammary tumours | | | | | | | | | Ovaries | | |
|---|---|---|---|---|---|---|---|---|---|---|---|---|---|---|
| | | | Adenocarcinoma | | | | | | | | | | | |
| 20-Methyl-cholan-threne-treated females | Animal number | Age at death in months | Small tubular | Papil-lary cystic | Irregu-lar tubular | Solid poly-gonal cell carci-noma | Intra-tubular carci-noma | Adeno-acan-thoma | Carci-noma simplex | Carcino-sarcoma | Com-bined weight of both the ovaries (mg.) | Sum of corpora lutea in both the ovaries | Granulosa cell hyperplasia |
| Breeders . | P 138 | 8·4 | — | (8) | — | — | — | (4) | — | — | 3·8 | 5 | Pseudofollicular. |
| | P 147 | 9·8 | 1 | — | — | — | — | — | — | — | 3·2 | 4 | — |
| | P 151 | 9·10 | — | — | — | 1 | — | (1)1 | — | — | 5·8 | 9 | Cribriform. |
| | P 166 | 10·6 | (1) | — | — | — | — | — | — | — | — | 19 | Pseudofollicular. |
| Forced breeders | P 146 | 8·13 | — | — | (1) | — | — | — | — | — | 2·8 | 9 | — |
| | P 142 | 8·15 | — | — | — | — | — | (1) | — | — | 11·9 | 14 | — |
| | P 149 | 8·16 | — | 1 | 1 | — | — | — | — | — | 1·7 | 9 | — |
| | P 173 | 8·18 | 1 | — | (1) | — | 1 | (1) | (1) | — | 12·9 | 13 | — |
| | P 170 | 9·10 | (1) | — | — | — | — | (1) | — | — | 27·9 | 11 | Tubular adenoma, follicular cyst. |
| | P 171 | 10·3 | (1) | — | — | — | — | (1) | — | — | 9·5 | 9 | Pseudofollicular. |
| Mated females which did not breed | P 155 | 9·4 | — | — | — | — | — | (1) | — | — | 4·7 | 13 | — |
| | P 156 | 9·4 | — | — | (1) | — | — | — | (1) | — | 0·9 | 0 | Diffuse leuteinization. |
| Forced breeders with pellets | P 218 | 6·13 | — | — | — | — | — | — | — | 1 | 8·2 | 9 | Pseudofollicular. |
| | P 302 | 9·4 | — | — | — | — | — | — | — | 1 | 5·8 | 6 | " |
| | P 251 | 9·9 | — | — | 1 | — | — | — | — | — | 10·4 | 4 | " |
| | P 257 | 9·23 | — | 2 | — | — | — | — | — | — | 9·0 | 7 | Diffuse luteinization. |
| | P 324 | 10·29 | (1) | — | — | — | — | (1) | — | — | 8·8 | 10 | Pseudofollicular. |
| Total . | 17 | — | 6 | 11 | 5 | 1 | 1 | 12 | 2 | 2 | 8·00 | 8·9 | 11/17 |

Figures in the bracket represent non-palpable lesions.

virgins also showed very few corpora lutea but a number of normal and anovular follicles. Granulosa cell hyperplasia was not seen in these females.

(ii) *Normal breeders.*—Unfortunately only a small number of normal control breeders of the same age-group was available for comparison. In six breeders, 7–10 months old and eight 10–12 months old the average number of corpora lutea was 3·4. The ovaries of the 20-methylcholanthrene-treated breeders of 7–10 months contained an average of 5·0 corpora lutea and many normal and anovular follicles (Fig. 6). Granulosa cell hyperplasia was observed in four of the six females.

(iii) *Forced breeders.*—The treated forced breeders developed on an average 4·6 well-formed corpora lutea. Granulosa cell hyperplasia was present in the ovaries of four females. No normal forced bred controls were maintained.

(iv) *Sterile females.*—Of the two females that failed to breed, one presented extensive diffuse luteinization in the ovaries, while in the other there were 6 to 7 functional corpora lutea (Fig. 8).

*Adrenal.*—The adrenals from all the groups, *i.e.* control and treated virgins, breeders and sterile females presented a normal histological picture. 20-Methylcholanthrene did not have any effect on the structure of the adrenal.

### *Subcutaneous Implantation of Pellets Containing* 20-*Methylcholanthrene*

*Breast pattern*

All the nine forced breeders implanted with 20-methylcholanthrene pellets showed good duct and acinar development.

*Breast tumours*

Seven of the nine forced breeders developed palpable tumours at the site of implantation of the pellets. With the exception of two fibrosarcomas of the subcutis the remainder were either adenocarcinomas, adenocanthomas or carcinosarcomas (Fig. 12).

Seven females showed tumours at the site of the pellet, two having multiple tumours. Two were fibrosarcomas of the subcutis, two were sarcomas with foci of tubular adenocarcinoma, one was a carcinosarcoma and three were adenocarcinomas of the mammary gland. One female showed two tumours which were not palpable and were classified as adenoacanthoma and adenocarcinoma respectively.

*Ovary*

These forced breeders showed a number of well-formed corpora lutea together with normal and anovular follicles. Early granulosa cell hyperplasia was seen in five of the nine females.

*Adrenals*

All the adrenals showed normal histology.

*Progeny*

Table IV compares the breeding behaviour and survival of litters of both control and 20-methylcholanthrene-treated breeders. The mortality was low in the progeny of the control as well as of the painted normal breeders.

Of ninety-two young born to the six painted breeders, forty-two survived. Ten females and four males from these are at present under observation. Five of these ten females are being maintained as forced breeders and have so far undergone four to six normal pregnancies. The remaining five females are virgins. The males appear normal and healthy.

TABLE IV.—*Breeding Behaviour of Control and 20-Methylcholanthrene-treated Female Parents*

| Type of parent | Total number of parents studied | Average number of pregnancies | Total number of young born | Average size of litter |
|---|---|---|---|---|
| Control breeders | 28 | 2·90 | 513 | 6·33 |
| Painted breeders | 6 | 3·00 | 92 | 5·16 |
| Painted forced breeders | 11 | 3·91 | 250 | 5·81 |
| Forced breeders with pellets | 9 | 3·77 | 195 | 5·73 |

### DISCUSSION

The albino strain L(P) is derived from a strain maintained in Paris designated L(C). This latter strain yielded a moderate incidence of mammary tumours in both virgins and breeders, and was thought to carry the milk factor. No mammary tumours have yet been observed in the L(P) strain among 29 virgins and 22 breeders ranging in age from 10–31 months (Table I). As no spontaneous mammary tumours were available for testing for the milk factor, an attempt was made to demonstrate acinar growth following a single application of oestradiol to the skin. This was unsuccessful, which suggests that the milk factor is not present (Pullinger, 1947).

A study of the breast structure of the L(P) strain showed that the duct system was well developed in virgins and breeders, but that acini were singularly deficient in virgins and disappeared from the breeders immediately after lactation. It was noted also that the number of corpora lutea in the ovary was of a low order, the largest number being observed in virgins of 5–6 months of age. In both virgins and breeders corpora lutea were deficient after 12 months of age. It is suggested that there is a causal relationship between the poorly developed breast and the low corpus luteum content of the ovary.

Assuming that this strain is a low breast cancer strain with a poorly developed breast, it seemed valuable to know whether tumours could be induced by chemical means. Two methods of application of 20-methylcholanthrene were used: continuous skin painting in benzene and subcutaneous implantation in paraffin wax. Both methods were successful in inducing tumours. A good yield was obtained in painted breeders, forced breeders and sterile mated females, as well as in implanted forced breeders. No tumours were obtained in eight continuously painted virgins surviving to 8 months of age. This result contrasts with that of Bonser (1954), who found that subcutaneous implantation of this chemical even for a limited period of time induced mammary tumours in IF virgins, and with those of Orr (1951) and Jull (1954), who found that skin applications of 20-methylcholanthrene in oil for a limited period was equally successful in inducing tumours in IF virgins. Marchant (1955), on the other hand, showed that pregnancy and lactation lowered the tumour yield in response to oily skin applications of 20-

methylcholanthrene to IF mice. An analysis of the number of corpora lutea (Table II) in control and carcinogen-treated mice showed that there was a tendency towards a higher corpus luteum content in the treated groups. As the corpus luteum content of the ovaries of non-treated forced breeders is not known, the question of the role played by the ovary in the chemical induction of the mammary tumours must remain uncertain. If further work should show that the corpora lutea are stimulated to function by the chemical, then it might well be that there is a causal relationship between the increased number of corpora lutea and the development of the mammary tumours.

The mammary tumours resulting from the methylcholanthrene treatment were of the usual varied type which is to be expected in chemically induced tumours. Twelve of the tumours were of a size which caused visible and palpable lumps, whereas the remaining 28 were only discovered at post mortem. There was a higher incidence of squamous change in the latter group, possibly due to their slower growth rate. There was no doubt that they were true tumours (Fig. 9, 11).

### SUMMARY

A new strain of albino mice, designated L(P), is described. It was derived from a Parisian strain inbred for 17 generations, and has been maintained in India during 14 generations of inbreeding. The breast of the virgin female has a well-developed duct system but only a poor acinar system ; acini disappear from breeders soon after lactation. Spontaneous mammary tumours have not been observed in 39 virgins and 28 breeders ranging in age from 7–31 months. The average number of corpora lutea per ovary is low, especially after the age of 6 months in virgins and 12 months in breeders.

Virgins and breeders were subjected to two forms of treatment by 20-methylcholanthrene : continuous cutaneous application of an 0·25 per cent benzene solution and subcutaneous implantation of paraffin wax pellets each containing 0·1 mg. of the carcinogen. Mammary carcinomas were induced by both methods of treatment. No tumours were obtained in 8 virgins dying at 7–8 months of age, but a good yield was obtained in painted breeders, forced breeders and mated females which failed to breed. Acinar development was also improved by the treatment.

20-Methylcholanthrene had no effect on the breeding behaviour of the L(P) mothers nor on the health of the progeny. Litters of normal size and number were born.

Grateful thanks are due to Dr. V. R. Khanolkar, for his encouragement and interest during the course of this work.

### REFERENCES

ANDERVONT, H. B. AND DUNN, T. B.—(1949–50) *J. nat. Cancer Inst.*, **10**, 895.
BONSER, G. M.—(1954) *J. Path. Bact.*, **68**, 531.
CLOUDMAN, A. M.—(1941) 'Biology of the Laboratory Mouse'. Philadelphia (The Blakiston Co.), p. 168.
FEKETE, E.—(1946) *Cancer Res.*, **6**, 263.
*Idem.*—(1953) *Anat. Rec.*, **117**, 93.

GARDNER, W. U., STRONG, L. C. AND SMITH, G. M.—(1939) *Amer. J. Cancer*, **37**, 510.
HOWELL, J. S., MARCHANT, J. AND ORR, J. W.—(1954) *Brit. J. Cancer*, **8**, 635.
JULL, J. W.—(1954) *J. Path. Bact.*, **68**, 547.
KHANOLKAR, V. R. AND RANADIVE, K. J.—*Ibid.*, **59**, 593.
MARCHANT, J.—(1955) *Ibid.*, **70**, 415.
ORR, J. W.—(1951) *Acta Un. int. Cancr.*, **1**, 294.
PULLINGER, B. D.—(1947) *Brit. J. Cancer*, **1**, 177.
TAYLOR, H. C. AND WALTMAN, C. A.—(1940) *Arch. Surg.*, **40**, 733.